# Do pictures influence memory and metamemory in Chinese vocabulary learning? Evidence from Russian and Colombian learners

**DOI:** 10.1371/journal.pone.0286824

**Published:** 2023-11-02

**Authors:** Beatriz Martín-Luengo, Zhimin Hu, Sara Cadavid, Karlos Luna

**Affiliations:** 1 Center for Cognition and Decision Making, Institute for Cognitive Neuroscience, HSE University, Moscow, Russia; 2 Department of Developmental Psychology and Socialisation, University of Padua, Padua, Italy; 3 School of Medicine and Health Sciences, Universidad del Rosario, Bogotá, Colombia; 4 Universidad Nacional de Colombia, Bogotá, Colombia; Universita degli Studi di Milano-Bicocca, ITALY

## Abstract

Despite the increasing interest in learning non-alphabetical languages such as Chinese, research about its learning process for alphabet users is scarce. Research conducted on Latin alphabet users on learning languages written in Latin alphabet, or on Chinese language learning in Chinese native speakers, users is undoubtedly useful but it does not inform about the peculiarities of leaning Chinese language by other alphabet users. Additionally, several authors have highlighted the need to inform and extend the current second language acquisition theories on the particular challenges of learning a language that uses another script. In this research we aim to contribute filling this research gap and studied the learning process of Chinese vocabulary by users of scripts different from Chinese. In particular, we examined the role of pictures and translations as learning aids for Chinese language vocabulary learning in participants familiarized with either one or two alphabetical scripts (different from the Chinese logographic script). One hundred thirteen participants studied word-aid pairs in different conditions: Hanzi (Chinese in Chinese characters)-picture; Pinyin (Chinese in Latin alphabet)-picture; Hanzi-translation; Pinyin-translation. Participants evaluated the future recallability of the words and their meanings (i.e., judgements of learning) and completed two recognition tests. Words in Pinyin and words-translation pairs were judged to be easier to remember than Hanzi and word-pictures pairs. Participants remembered the meaning of words written in Hanzi better than in Pinyin, and word-translations pairs better than pictures, but they were more confident about word-picture pairs. These results suggest that pictures boost confidence in learning Chinese, but do not affect performance. These findings suggest that while pictures may boost confidence in learning Chinese, they may not necessarily lead to better performance. Our study provides valuable insights into the interaction of learning aids and writing system in (meta)memory during vocabulary acquisition.

## Introduction

In the current globalized world, it is increasingly common to learn new languages that use unfamiliar scripts. However, research on the processes involved in learning languages with unfamiliar scripts is scarce, despite their obvious relevance to, for example, developing effective educational materials. The last two decades have witnessed an exponential number of people learning Mandarin Chinese as a second or foreign language (CSL/CFL) [1, 2]. Hereafter, we refer to Mandarin Chinese when we mention the Chinese language. The Chinese language uses a logographic script, which makes it particularly challenging for non-logographic script users learning Chinese. Yet, the current theories on second language acquisition (SLA) are mainly based on alphabetical languages and would need to be extended to integrate the specific challenges related to learning languages with logographic scripts [1, 3, 4]. In this line, a recent meta-analysis highlighted the increasing demand for learning Chinese during the last 30 years and the need for CFL research [1]. In the present research, we aim to contribute to fill this research gap and study the learning process of a logographic script (i.e., Chinese) for non-logographic script users (i.e., Cyrillic and Latin alphabets users).

An important, yet sometimes overlooked, process during language acquisition is the monitoring of the learning process. Monitoring is part of the wider concept of metamemory (itself part of metacognition) and refers to the evaluation of the quality of the encoding processes. Successful monitoring during learning processes allows the implementation of strategies that may maximize the acquisition of new knowledge [5–8]. However, a number of studies have shown that many different variables can distort monitoring processes [9–13] and, consequently, hinder the learning process. Learning languages implies a series of processes that have accumulated a large body of research over the past decades. However, the research focused on the ongoing metamemory processes during language learning is underrepresented: “it can still be claimed that metacognition has not yet been recognized as an integral part of language learning and teaching by as many researchers and scholars as desired” [14, p. 1]. In this sense, to our knowledge, only one recent study was deemed worthy of attention [15], but in that study alphabet users learned another alphabethical language. Here, we will pay special attention to metamemory processes to complement memory measures of language acquisition in a logographic script.

As previously stated, many speakers whose native writing systems are alphabetic (i.e., Latin and Cyrillic) show a growing interest in learning Mandarin Chinese, whose writing system is based on characters that represent individual words or morphemes (i.e., logographic script; [16]). This trend demands specific research that should unveil to what extent, for these learners, learning a logographic language (e.g., Chinese) differs from learning other alphabetic languages (e.g., English). The proposed models on visual word recognition in different orthographies [17] are mainly based on foreign vocabulary learning studies of languages with alphabetic orthographies. Extending such models to learning languages with unfamiliar scripts mandates more targeted scientific evidence. More importantly, existent models need to consider metacognitive measurements as different scripts present idiosyncratic cognitive and metacognitive challenges for learning. For instance, Chinese characters have a high visual complexity for processing [18] and generate a greater visual load than other writing systems [19]. Given that both cognitive and metacognitive operations draw from the same pool of resources and both are crucial for successful learning [20], it is likely that the Chinese script affects metacognitive processes in learners with an alphabetic background. Thus, in the present research we aimed to investigate memory and metamemory processes in Mandarin Chinese vocabulary language learners familiar with just one (Colombian: Latin) and two alphabets (Russian: Cyrillic and Latin). Additionally, since vocabulary learning requires semantic pairing, we specifically compared the memory and metamemory measurements in two traditional learning aids: pictures and translations.

This interdisciplinary study focuses on several topics linked with different areas and tests two main objectives:

- To study the memory and metamemory processes of learning a language with a logographic script for alphabet-script users.
- To further understand the effectiveness of different learning aids on second-language learning, with an interest in both metamemory and memory processes.

These objectives are equally relevant to the present study. Thus, we review the pertinent literature for each objective but also try to integrate the results to get a more comprehensive understanding of the learning process under investigation.

### Peculiarities of Chinese language to different types of learners

The Chinese language script, Hanzi, is composed of radicals that form characters. The combination of characters forms words. However, Hanzi does not reflect direct phonemic representations of sounds like an alphabet. This lack of apparent phonetic cues makes many people without previous exposure to Chinese see Hanzi as drawings or even link the words written in Hanzi with familiar visual representations. Indeed, past research [21] found that while some English-speaking students simply viewed each Hanzi character as a picture, others often encoded compound Hanzi characters using figuratively similar letter combinations, such as “EP” for “印” or “KON” for “糊”.

Chinese can also be written in Pinyin, the official Romanized script for standard Mandarin Chinese. Pinyin makes use of the Latin alphabet and tone diacritics to represent pronunciation. Thus, Pinyin can essentially be considered an alphabetic orthography of Chinese. Research has shown that Pinyin helps young Chinese children with sentence reading in Hanzi [22] and in the comprehension of texts with a large amount of unfamiliar Hanzi [23]. Since the phonological channel in the working memory is dominant in vocabulary acquisition and phonological awareness is transferable across alphabets [24, 25], learning words written in Pinyin should be easier than in Hanzi. The capacity to read and repeat words, by maintaining them in the phonological loop in the working memory, has been proven to facilitate their transference to the semantic long-term memory system (for a recent review on the topic see, [26]). Hence, vocabulary learning should be easier for words that are phonologically accessible, that is, those words that the learner can read. In that case, the learner would be using both routes, phonological and orthographic (for more information on the dual-route models of reading, see [27, 28]. However, a learner with limited phonological knowledge of the script could only use the orthographic route, resulting in a decrease in the number of cues connected to the new word because there is only one processing route during encoding, and this will complicate its later retrieval [29]. Therefore, Chinese-Language learners who are used to a different script (i.e., Latin, Cyrillic, Arabic, Bangla) face challenges that need to be properly understood.

To the best of our knowledge, only one recently published study has approached the challenge that CFL (Chinese as Foreign Language) learners face when they are not used to Hanzi. Classical classifications split CFL learners in two groups: Students already familiarized with Hanzi (e.g., Japanese or Koreans) and the so-called alphabetic learners who are not familiar with Hanzi, where English, Arabic, and another native writing system users are put together despite their writing system considerably differ [3]. To study the validity of such classification, a recent study [3] examined Hanzi writing, reading, and phonological skills in pre-intermediate and intermediate CFL adult learners who were native speakers of Arabic or English. Arabic L1 CFL learners performed better than English L1 CFL learners, highlighting the need for specific research on CFL with different L1 learners.

However, it remains under question whether knowing more than one script within a writing system also influences Chinese learning. There are reasons to believe that one-script users and two-script users may have different skills in tackling Chinese-language learning. For example, in relation to monitoring the learning processes, people used to different scripts, like Russians, already know they can manage Cyrillic and Latin scripts. In contrast, one-script-users like Colombians (i.e., only Latin script users) do not have that experience and may undervalue their capabilities to learn another script or writing system. Moreover, these advantages of multiple-script users may show memory advantages over one-script users. If that were the case, exclusive users of Latin alphabet might have more difficulty recognizing a word written in Hanzi than regular users of two different scripts. The use of two or more scripts may provide multiple-script users with a different mindset towards the challenge of learning a new script and give them an advantage, because they may have developed language use strategies (i.e., the capability of switching between two different scripts). Since using one or more scripts might vary the perception and capabilities towards learning a language in a new script, in this research we studied how two-script users (Russians) and one-script users (Colombians) learn Chinese vocabulary differently. We did not find other studies in which Chinese vocabulary learning is examined under the lens of the number of scripts used by the participants, nor did we encounter studies that tested learners unfamiliar with a logographic language.

### Vocabulary learning aids

Vocabulary learning is a different process for first-language and late-second or foreign-language learners [30]. In a very simplistic way, one of the main differences in the vocabulary learning process for first- and foreign-language learners is that first-language learners have to learn forms and meanings, whereas foreign-language learners already know the meanings. Despite much research supporting that an early age of acquisition of a second (or more) language is crucial to achieve a high proficiency [31, 32], learning a foreign language in the adulthood may also have some advantages for learning.

In this research, we focus on foreign-language vocabulary learning in the adulthood. Adults have some characteristics that make them more permeable to rely on aids during the learning processes. For example, they already know how languages work, and possess cognitive tools that can foster vocabulary learning and a trained memory capacity that can compensate for the drawbacks of learning a new language out of the so-called critical period [33, 34].

The most widely used (and investigated) learning aids for vocabulary learning are pictures and translations, also termed picture-word associations and word-word associations, respectively [35–38]. The picture-word association consists of pairing words in the new language with a descriptive picture that depicts the meaning. In this case, vocabulary learning is grounded in semantics (i.e., the conveyed meaning of the picture). As for the word-word association, the word in the new language is presented along with its definition or translation in the learner’s language. Vocabulary learning here is grounded in the known lexicon. Both aids have advantages and limitations. For example, pictures are often not suitable for abstract words, as they may oversimplify or distort the nuances and complexities of abstract concepts. However, conveying abstract concepts is not a problem for translations in the learners’ native language. Instead, translations can hinder learning due to the lexical competition between the native and the new language or the interference from a similar lexicon but a different meaning in the new language [39]. Still, both methods are widely used in textbooks as means of learning vocabulary in another language [30]. In spite of the prevalence of these two learning aids in language learning, few studies have focused on their unique effects on both memory and metamemory processes in learning written Chinese vocabulary from alphabet users’ perspective. We hereafter review relevant research in both realms.

### Metamemory and vocabulary learning aids

Metamemory processes can be examined during encoding with judgments of learning (JOLs), which are monitoring metacognitive judgments about the future recallability of a piece of information [40]. The formation of JOLs has been explained both in terms of the processing fluency experienced by participants [41, 42] and the beliefs they already have about any characteristic of the examined material [43, 44]. These monitoring judgments have been extensively studied in educational settings and are informative about the learning process [45]. However, some variables, such as the presence of pictures, can misalign JOLs and memory. For example, one study showed that people judged their comprehension of a text more positively when the text was accompanied by photos, though the presence of these photos did not affect actual learning [46]. Similarly, explanatory pictures and diagrams inserted within a scientific text led students to think that the text was easier to understand, although there were no differences in actual text comprehension [47]. These results suggest that images and graphics produce impressions of learning or comprehension, but they do not necessarily improve learning [48].

When new words in a second language are paired with pictures, JOLs show similar results. For instance, a previous research [49] found a misleading effect of pictures representing the to-be-studied word in foreign vocabulary learning (i.e., picture-word association) when compared with the presentation of translations (i.e., word-word association). Specifically, participants provided higher JOLs for the words paired with pictures than with translations, whereas learning was not affected by the type of aid. Pictures created an illusion of learning because they increased the feeling of processing fluency, as salient stimuli like pictures are more easily processed for memorization [49]. This result is indeed consistent with research in processing fluency that shows that higher metacognitive judgments are attributed to more fluently processed stimuli such as high-frequency words [50, 51] or clearer fonts [52].

Recently, a new perspective was suggested on the misleading effect of pictures in foreign language vocabulary learning [53]. These authors suggested that metacognitive judgments in foreign language learning are driven more by students’ *beliefs* about learning rather than by processing fluency. In their research, native English-speaking participants went through a self-paced study phase of Swahili words, either paired with pictures or with English translations, and later completed a cued-recall task. Their results showed that participants in the picture-paired condition gave higher judgments of learning regardless of whether the judgments were made immediately or after a delay. Additionally, they found that participants estimated pictures to be more effective in facilitating memory, regardless of their previous learning experience. In that research, the authors reasoned that the a priori assumption of the superiority of pictorial cues undermined the effective processing of the words, leading to no observable benefits in learning outcomes with pictures [53].

In sum, the research reviewed above shows that JOLs tend to be higher when the new vocabulary is presented with pictures, an effect that may be due to participants’ processing fluency or, as recently suggested, beliefs. However, these studies used alphabetic languages, and therefore transferring their findings to logographic languages such as Chinese is not straightforward and mandates rigorous scientific evidence.

There are reasons to think that the cognitive mechanisms involved in learning Chinese may differ from those implied in learning alphabetic languages due to its relatively high cognitive load for alphabet users [54]. This idea is grounded on evidence that, in certain situations such as under high cognitive load, participants disregard previous beliefs [55]. Thus, for a Chinese-language learner whose native language is alphabetic, processing written words in Chinese would be difficult, and that extra difficulty may lead them to disregard any existing belief about the benefit of pictures for learning. Similarly, when confronted with the difficult task of learning a new Chinese word, these learners may have more free resources to process Chinese words that are paired with translations (vs. pictures) due to more processing fluency of translations in their native language [39]. Thus, they may estimate that translations are better than pictures when learning Chinese. Moreover, some work on vocabulary learning where no JOLs were required showed the superiority of translations over pictures for alphabetic languages, such as Italian [37], French [56], and Swahili ([58], Exp. 1–3). In sum, the current evidence is not sufficient in clarifying the role of these two learning aids for learning Chinese from a metacognitive perspective.

### Memory and vocabulary learning aids

In vocabulary learning, there is much research comparing the efficacy of pictures vs. translations, but it has shown mixed results [35–38, 57–61]. For example, the earliest works [e.g., 58] compared foreign vocabulary learning by pairing pictures and translations, and their results suggested that pictorial cues sped up vocabulary memorization. According to other authors [62], the difference between pictures and translations is that pictures contribute to better results in memory by allowing visual encoding of words in addition to phonological encoding. Cross-linguistic studies on Indonesian students learning English [60], college-aged English-speakers learning French [59], and young Japanese learning English [61, 62] reported similar results pointing to pictures enhancing the learning of new vocabulary. On the other hand, there is also evidence in support that translations were more effective than pictures in the case of Dutch speakers learning Italian [37] and Chinese speakers learning French [56].

Despite the extensive research on the use of aids in foreign vocabulary learning, to the authors’ knowledge, this has been primarily studied in learning alphabetic languages [37, 56, 61], except for the recent work [63]. These authors tested, in alphabet users and Chinese users, the effect of radical marking and stroke order animation in learning Hanzi for words presented with definitions or with drawings. These aids helped Chinese native learners to improve their Hanzi learning, but in alphabetic native users the additional information conveyed by the animation interfered negatively with Hanzi learning. As the authors pointed out, the fact that the alphabetical native CFL learners obtained better memory results for Hanzi aided with definitions and not with the drawings is an indication that, for them, pictures induced an overload of information to process. Their finding is in line with the cognitive load theory [64], that is, the increased cognitive load of learning a more difficult script may make participants appreciate more the ease of processing translations (vs. pictures) as learning aids. Hence, our research aims to further clarify this relevant point in the literature of foreign vocabulary learning concerning the use of both learning aids for learning Chinese.

### The present research

We conducted a study to investigate the metamemory and memory effects of learning aids (pictures vs. translations) for Chinese vocabulary learning (Hanzi vs. Pinyin) by users of one or two scripts (Latin vs. Latin and Cyrillic). As per metamemory, we expected that (1) words in Pinyin would be rated with higher JOLs than words in Hanzi. We cast this prediction because both one- and two-script participants use the Latin alphabet, and they may feel that words in Pinyin are more familiar and easier to learn. We also expected that (2) a learning aid would affect JOLs for words in Hanzi but not in Pinyin. Specifically, we anticipated that, in the Hanzi-translation condition, participants would show higher JOLs than in the Hanzi-picture condition. The high complexity of Hanzi characters could overload participants’ visual processes, making them experience processing disfluency when paired with pictures and rely more on a phonologically available aid (i.e., translations). Past research suggested increased processing fluency for information presented alongside pictures, but that research was conducted with Latin alphabet users learning a new language in the Latin alphabet. It is plausible that the participants’ lack of experience with logographic scripts may reverse the feelings of fluency triggered by pictures and in turn favor translations. We also expected that (3) two-script users would rate Hanzi words with higher JOLs than one-script users. This difference would emerge due to either two-script users undervaluing the complexity of the Hanzi script derived from an illusory sense of multi-script command or one-script users underestimating their capability to learn a new script.

Turning to memory, we also expected (4) general better memory for words in Pinyin than Hanzi, consistent with JOLs, because both one and two-script users use the Latin alphabet, and this allows them to use both the orthographic and the phonological routes to encode and retrieve Pinyin words. As for the effect of learning aid, due to the discrepancy in the efficacy of the learning aids in vocabulary learning and the differences between past research with alphabetical and Chinese languages, we tentatively expected (5) no differences in memory for words paired with pictures or translations, or a better performance of translations over pictures, but there is not much research supporting this hypothesis. Similarly, (6) we did not expect differences in memory per the number of familiar scripts by user. That is, our predictions for the effect of learning aid and number of scripts in memory and metamemory differ because we expected they would affect metamemory but not memory.

To test these hypotheses, one- and two-script participants were presented with Chinese words written in either Pinyin or Hanzi, paired with either pictures or translations. During encoding, participants made JOLs for each pair, and then completed two memory tests: a Chinese word recognition test and a Chinese word-learning aid pair recognition test. Finally, participants answered questions about their beliefs regarding the effect of pictures and translations in learning Chinese.

## Method

### Participants and research design

This study has a 2 (type of script: Hanzi, Pinyin) x 2 (aid: picture, translation) x 2 (number of scripts: one-script user, two-scripts user) design with type of script and aid manipulated within-participants. Fifty-six Russian native speakers (42 female; age *M* = 25.96; *SD* = 3.44) and fifty-seven Colombian Spanish native speakers (40 female; *M* = 19.79; *SD* = 2.69) participated in the online experiment. Russian participants had a good command of two scripts (i.e., Cyrillic and Latin), whereas the Colombians commanded only one script (i.e., Latin). All the participants were university students, and the average age of both samples together was 21.88 years old (*SD* = 3.72). We did not compute an a priori power analysis, but computations with WebPower [65] showed that with alpha = .05 and power = .80, a sample of *n* = 113 could detect effects of *d* = 0.31 for the within effects and two-way within-between interactions, and of *d* = 0.27 for the between variable. Thus, our experiment was powered enough to detect small effects. WebPower does not compute power for three-way interaction, which was not included in our original hypotheses. The Russian participants received 250 rubles after the experiment and Colombian participants participated without any monetary reward. We used a 2 (type of script: Hanzi, Pinyin) x 2 (aid: picture, translation) x 2 (number of scripts: one-script user, two-scripts user) mixed design with type of script and aid manipulated within-participants.

### Materials

To guarantee that all the Chinese characters had similar visual complexity, we first selected Chinese characters by limiting their stroke number to between 4 and 6. Out of the eligible Chinese characters, we only used concrete nouns to allow more pictorial representability. In total, we selected 95 Chinese characters from the *Table of General Standard Chinese Characters* (http://hanzidb.org/character-list/general-standard). Then, we ranked the Chinese words according to the word frequency of their Russian translations (http://dict.ruslang.ru/freq.php). Infrequent words were eliminated from the list (frequency per million, fpm < 10). Finally, we obtained 93 words (fpm, *M* = 132.22).

To select the pictures, we conducted a normative study in which we presented each word in the native language of the participant and three black-and-white pictures collected from various sources on the Internet. We did not use picture databases already available because they did not cover the entire sample of selected words. Four native Russian speakers with no prior experience in art education were asked to first identify the item in the pictures, and then to rate the representability of the picture for the item on a scale of 1 (minimal representability) to 5 (maximal representability). The final set of pictures was chosen based on the following criteria: the number of identifications, the sum and standard deviation of the representability ratings, and the pixel complexity of the picture. After the screening, we ended up with a pool of 89 word-picture pairs. Forty words (fpm, *M* = 204.57) were used in the actual experiment.

**Fig 1:**
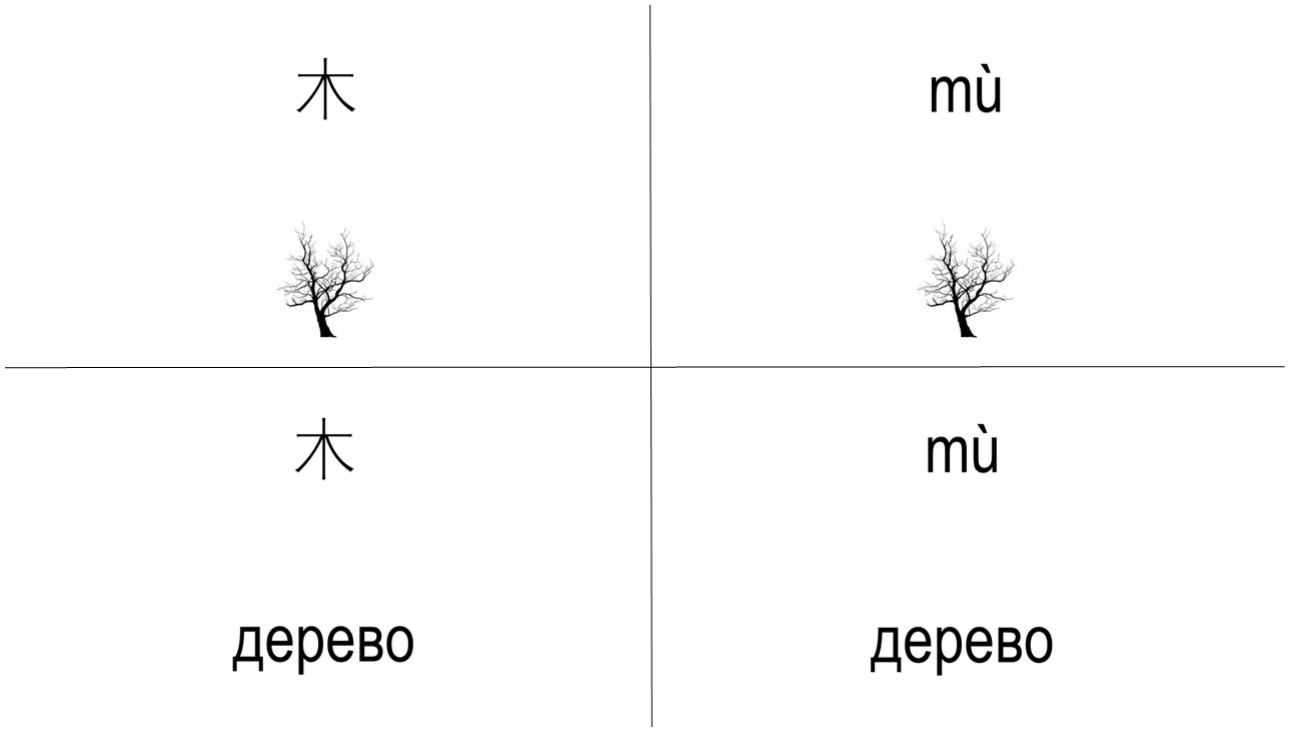
Representations of the four conditions in the experiment for Russian participants. Note. Upper-left quadrant: the word “tree” written in Hanzi and its picture aid. Lower-left quadrant: the word “tree” written in Hanzi and its translation into Russian written in Cyrillic. Upper-right quadrant: the word “tree” written in Pinyin and its picture aid. Lower-right quadrant: the word “tree” written in Pinyin and its translation into Russian written in Cyrillic.

Combining the two within-participants variables of type of script and aid, we obtained four conditions (see Fig 1): Hanzi-picture, Hanzi-translation, Pinyin-picture, and Pinyin-translation. There were two versions of the same experiment, one in Russian for the Russian participants (two-script condition), and another in Spanish for the Colombian participants (one-script condition). The materials were first developed in Russian and then translated into Spanish for the Colombian sample. Eight Colombian Spanish-native speakers evaluated the translations and the suitability of the selected words and pictures. All the participants showed high agreement on the frequency of the selected words and on the representability of the pictures in relation to their Russian counterparts.

### Procedure

The procedure of this study was approved by the Ethics Committee of HSE, Moscow. We conducted an online experiment programmed in Gorilla Experiment Builder [66]. The average duration of the entire experiment was approximately 25 minutes. First, we asked participants if they had previous knowledge of Chinese and/or Japanese, and had previous artistic training. By answering these two questions, the prior level of command or familiarity with logographic scripts was controlled in both samples. As mentioned above, non-natives may see Hanzi characters as pictures or drawings. Thus, the artistic training question sought to control whether some participants could have an advantage when processing the characters.

**Fig 2:**
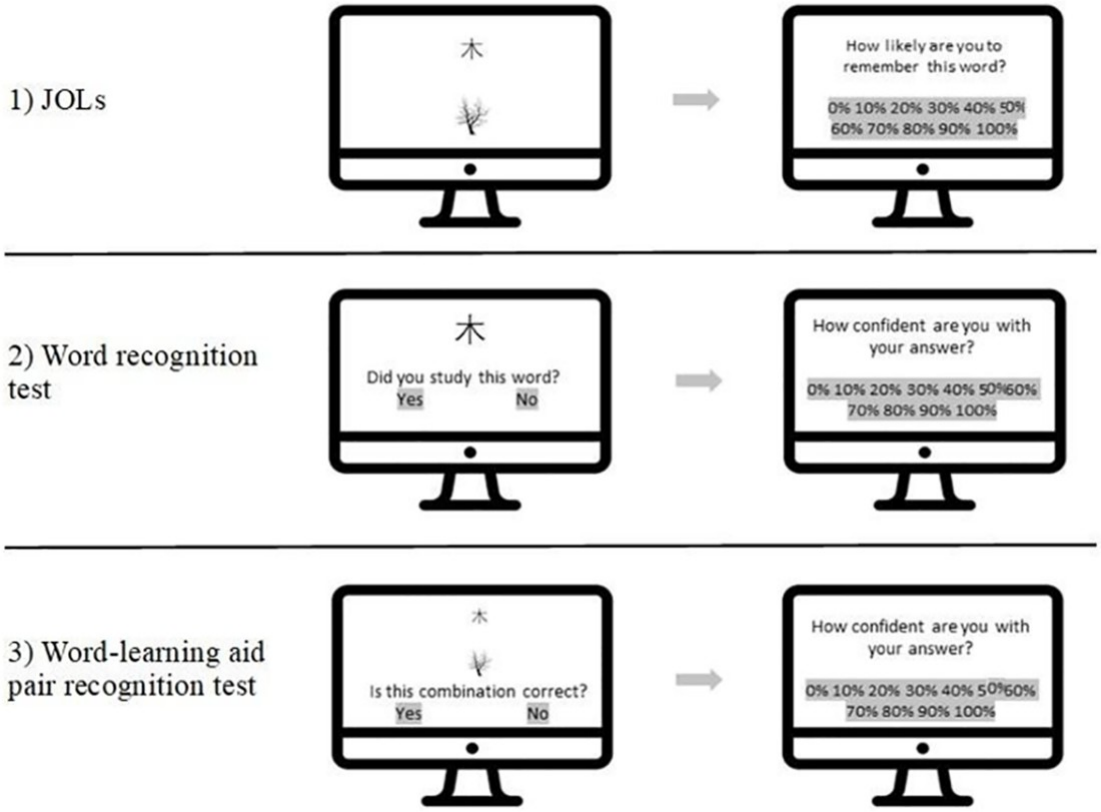
Experimental procedure (Translated into English).

Only if both questions were answered negatively, were participants invited to enroll in the experiment and were given a consent form. Upon agreement, they provided basic demographics and were then presented with the instructions and experimental procedure (see Fig 2). The instructions stated that 40 Chinese words written in either Hanzi or Pinyin would be presented to them for five seconds each, with their meaning either represented by a picture or a translation. Participants were encouraged to pay attention to the words because they would have to remember them later. Prior to the task, participants completed a short practice session of four study trials and one trial for each of the two recognition tests. The words and pictures in the practice sessions were not used in the experiment.

After the training session, participants were instructed to click on the “Start” button on the screen to begin the experiment. First, a fixation point was presented in the center of the screen for one second, followed by the word-aid pair in the center of the screen for five seconds. The words were always displayed above the aid. The words in Russian for the Russian participants, Spanish for the Colombian participants, and Pinyin for all participants were presented in Arial narrow font, while words in Hanzi were written in Dengxian light font. After each word-aid pair, a different screen appeared automatically after five seconds, where participants rated the likelihood of recalling the words later (JOL) by selecting the appropriate button on a Likert scale from 0% to 100% in deciles. Participants were instructed to select 0% if they were certain that they would not remember the word later, and to select 100% if they were completely certain that they would remember it.

After studying the 40 word-aid pairs, participants completed three different filler tasks involving numbers. In the first numerical task, participants were shown an initial number and were asked to make several sequential mental additions or subtractions. They had four seconds for each operation. After six operations, participants were instructed to type in the final number. Then they clicked on the “Next” button to complete two similar trials. In the second numerical task, participants were shown an initial number, and then they were asked to make mental comparisons with numbers that appeared on subsequent screens. Participants selected their answer by clicking the “Bigger” or “Smaller” buttons. The screen changed automatically as soon as participants made their selection. After comparing the initial number with six other numbers, they completed two similar trials. In the third numerical task, participants were asked to find the missing number in a series. The series of numbers were placed in random places on the screen and varied in size. They were instructed to select the missing number by clicking on the appropriate button. Participants completed three trials of this task and spent an average of 3.5 minutes completing all the filler tasks.

Subsequently, participants completed two recognition memory tests. In the first test, only words written in Chinese, either Hanzi or Pinyin, were displayed in the center of the screen. Below the word, participants were instructed to select “Yes” or “No” to indicate whether they had previously studied the word or not. Half of the words (20) had been presented and the other half (20) were new. Presented words were always shown in the same script as in the study phase. As soon as participants made their selection, a different screen appeared, where they rated the confidence of their answer by selecting the appropriate button on a scale from 0% (not confident at all) to 100% (completely confident) in deciles. After that, participants completed a second recognition test for the word-aid pairs. Half of the word-aid pairs were correct pairs presented in the study session (20), while the other half (20) were incorrect pairs-matches. Below the word-aid pair, participants were instructed to select “Yes” or “No” to indicate whether the word-aid pairs were correct or not, and on a different screen they indicated the confidence in their answers on a scale from 0% to 100% in deciles as in the previous task. The words that appeared in the first test were not used in the second test, as in either experiment only half of the Chinese words had been studied. The words used on each test were counterbalanced, but we did not counterbalance the order of the tests to avoid interference (i.e., all participants did the yes/no recognition test first and then the word-aid pairs recognition test). If participants were presented with the word-aid pairs recognition test first, they could have encoded new information (foils) as originally presented, and they could have refreshed their memories for the original words. These two situations can potentially influence the results of the yes/no recognition test.

At the end of the experiment, participants completed a *beliefs* measure. First, participants read a short text explaining that future Russian/Colombian volunteers for the 2022 Winter Olympics in China had completed the same experiment (the study took place before the Olympics). Then, they were asked to predict the volunteers’ learning performance to measure participants’ beliefs about learning Chinese vocabulary. Participants were presented with a word-aid pair in one of the conditions of the experiment (e.g., Hanzi-picture). Below the pair, there was a scale with 11 numerical labels ranging from 0 to 10. Participants were instructed to select 0 if they thought the volunteers had not remembered any word in that particular condition, and to select 10 if they thought volunteers had remembered all words in that condition. The participants completed this task four times, one for each condition.

## Results

### Statistical tests

We first performed outlier detection [67] on the reaction time of the recognition tests, and eliminated a Colombian participant for extremely fast reaction time. For the remaining data of the 112 participants, we conducted 2 (type of script: Hanzi, Pinyin) x 2 (aid: picture, translation) x 2 (number of scripts: one, two) mixed ANOVAs with type of script and aid manipulated within-participants. When appropriate, pairwise comparisons using Student’s *t* test were calculated. Bearing in mind the scarce research on the topics under investigation, we conducted a number of analyses following both a hypothesis-driven and data-driven statistical strategies to better grasp the memory and metamemory consequences of our manipulations. We report analyses for the following measurements: JOLs, word and word-aid recognition, and beliefs. For word and word-aid recognition, we applied SDT analyses on hits, false alarms, A′ [68] for accuracy, and B′′_D_ [69] for response criterion. In addition, we also analyzed confidence on hits and false alarms, but results were uninformative. Alpha for all analyses was set to .05. We report partial eta-squared (η_p_^2^) as the effect size measure for the ANOVA, and unbiased Cohen’s *d* (equivalent to Hedges’ *g*, [70]) for *t*-tests. To help readers navigate the results, we added brief reminders of the expected outcomes in each section.

### Judgments of learning

See Tables 1 and 2 for descriptive statistics and full results from the ANOVA for JOLs. We expected higher JOLs for Pinyin than for Hanzi (H1) and, for words in Hanzi, higher JOLs for translations than for pictures (H2). We also expected that one-script participants would underrate their capability to learn words in a new script and thus expected that two-script participants would rate Hanzi words with higher JOLs than one-script users (H3).

**Table 1:** Means (Standard deviations) for type of script, aid, and country in JOLs.

	*Number of Scripts*			
	Two-script		One-script	
***Script / Aid***	Pictures	Translations	Pictures	Translations
Hanzi	45.14 (17.19)	45.93 (17.76)	44.80 (19.50)	44.09 (19.41)
Pinyin	46.98 (17.79)	48.55 (18.50)	52.89 (17.25)	50.21 (18.07)

**Table 2:** Full results from the ANOVAs for JOLs and beliefs.

JOLs	Type of Script	*F*(1, 110) = 24.590, *p* < .001, η_p_^2^ = .183 ***
	Aid	*F*(1, 110) = 0.188, *p* = .665, η_p_^2^ = .002
	Number of Scripts	*F*(1, 110) = 0.181, *p* = .671, η_p_^2^ = .002
	Type of Script*Aid	*F*(1, 110) = 0.160, *p* = .690, η_p_^2^ = .001
	Type of Script*Number of Scripts	*F*(1, 110) = 6.700, *p* = .011, η_p_^2^ = .057 *
	Aid*Number of Scripts	F(1, 110) = 5.801, *p* = .018, η_p_^2^ = .050 *
	Type of Script*Aid*Number of Scripts	F(1, 110) = 0.870, *p* = .353, η_p_^2^ = .008
Beliefs	Type of Script	*F*(1, 110) = 1.147, *p* = .287, η_p_^2^ = .010
	Aid	*F*(1, 110) = 1.064, *p* = .305, η_p_^2^ = .002
	Number of Scripts	*F*(1, 110) = 3.758, *p* = .055, η_p_^2^ = .033
	Type of Script*Aid	*F*(1, 110) = 0.129, *p* = .720, η_p_^2^ = .001
	Type of Script*Number of Scripts	*F*(1, 110) = 0.184, *p* = .669, η_p_^2^ = .002
	Aid*Number of Scripts	*F*(1, 110) = 2.220, *p* = .139, η_p_^2^ = .020
	Type of Script*Aid*Number of Scripts	*F*(1, 110) = 0.920, *p* = .340, η_p_^2^ = .008

*Note*:

* *p* < .05;

** *p* < .01;

*** *p* < .001

**Fig 3:**
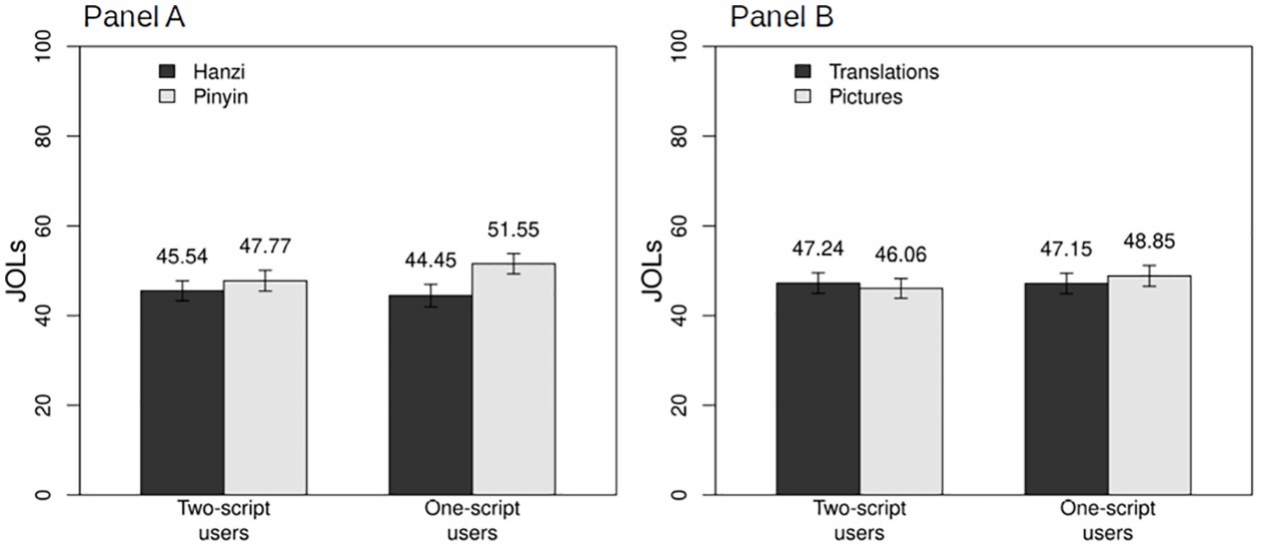
Interactions of Judgments of Learning for Number of Scripts X Type of Script (Panel A), and Number of Scripts X Aid (Panel B).

As expected, participants assigned higher JOLs to words written in Pinyin (*M* = 49.66, *SD* = 17.19) than in Hanzi (*M* = 44.99, *SD* = 17.76). There were no differences in aid and number of scripts. Two interactions were significant, number of scripts by type of script, and number of scripts by aid (see Fig 3).

In the interaction between number of scripts and type of script, one-script participants rated words in Pinyin with higher JOLs (*M* = 51.55, *SD* = 16.92) than those in Hanzi (*M* = 44.45, *SD* = 18.93), *t*(55) = 4.88, *p* < .001, *d* = 0.40, whereas there was no difference for two-scripts participants (for Hanzi *M* = 45.54, *SD* = 16.65; for Pinyin *M* = 47.77, *SD* = 17.40), *t*(55) = 1.87, *p* = .067, *d* = 0.13. Our prediction for this interaction was partially confirmed. We expected that two-script participants would trust in their capability for learning words in a new script because they can already manage two, and we did not find differences in JOLs for two-script users in relation to which script they were learning. At the same time, one-script participants rated Pinyin as easier to learn than Hanzi. A possible explanation for this pattern of results might stem from the availability of the native script across the different conditions. When learning Pinyin words, one-script users were exposed to their usual script (i.e., Latin), so they could have automatically triggered both visual and auditory encoding. However, when faced with Pinyin words, two-script users (Russian natives) were more used to Cyrillic, and thus were dealing with a second-language script. Encoding words in a second-language script might have supposed an extra cost of processing for two-script participants, which may have prevented them from judging Pinyin words as more likely to be remembered.

The interaction between number of scripts and aid showed that one-script participants rated words paired with pictures as more memorable (*M* = 48.85, *SD* = 17.43) than words paired with translations (*M* = 47.15, *SD* = 17.23), *t*(55) = 2.29, *p* = .026, *d* = 0.10, but there was no difference for two-script participants (for pictures *M* = 46.06, *SD* = 16.43; for translations *M* = 47.24, *SD* = 17.16), *t*(55) = 1.26, *p* = .213, *d* = 0.07. One-script participants considered pictures as more helpful but two-script participants did not consider that any of the aids would help them to recall better. Our prediction that learning aid would affect words in Hanzi was not confirmed.

### Memory

We expected overall better memory for Pinyin than Hanzi words in both recognition tests (H4). We did not expect memory differences by aid (H5) or number of scripts (H6).

#### Memory in the word recognition test

The word recognition test was composed exclusively of words presented in Hanzi or Pinyin, without any learning aid. See Table 3 for descriptive statistics and Table 4 for the full outcomes of the ANOVAs.

**Table 3:** Means (Standard deviations) for the proportions of hits, false alarms, A′ and B′′_D_ for the word recognition test.

		Two-Scripts Participants		One-Script Participants	
		Picture	Translation	Picture	Translation
Hits	Hanzi	.72 (.19)	.71 (.17)	.71 (.17)	.74 (.18)
	Pinyin	.71 (.18)	.74 (.14)	.67 (.16)	.71 (.16)
False alarms	Hanzi	.23 (.14)	.26 (.17)	.24 (.15)	.28 (.16)
	Pinyin	.28 (.16)	.25 (.17)	.26 (.18)	.26 (.16)
*A′*	Hanzi	.83 (.10)	.81 (.12)	.81 (.11)	.80 (.14)
	Pinyin	.79 (.12)	.82 (.11)	.77 (.14)	.81 (.09)
*B′′*_*D*_	Hanzi	.05 (.58)	.08 (.54)	.13 (.51)	-.05 (.51)
	Pinyin	-.01 (.56)	.03 (.51)	.17 (.48)	.06 (.53)

**Table 4:** Full results from the ANOVAs for the word recognition test.

Hits	Type of Script	*F*(1, 110) = 0.307, *p* = .580, η_p_^2^ = .003
	Aid	*F*(1, 110) = 4.010, *p* = .048, η_p_^2^ = .035 *
	Number of Scripts	*F*(1, 110) = 0.597, *p* = .441, η_p_^2^ = .005
	Type of Script*Aid	*F*(1, 110) = 0.889, *p* = .348, η_p_^2^ = .008
	Type of Script*Number of Scripts	*F*(1, 110) = 1.137, *p* = .580, η_p_^2^ = .003
	Aid*Number of Scripts	*F*(1, 110) = 1.064, *p* = .305, η_p_^2^ = .010
	Type of Script*Aid*Number of Scripts	*F*(1, 110) = 0.372, *p* = .543, η_p_^2^ = .003
False alarms	Type of Script	*F*(1, 110) = 0.525, *p* = .470, η_p_^2^ = .005
	Aid	*F*(1, 110) = 0.289, *p* = .592, η_p_^2^ = .003
	Number of Scripts	*F*(1, 110) = 0.101, *p* = .751, η_p_^2^ = .000
	Type of Script*Aid	*F*(1, 110) = 3.097, *p* = .081, η_p_^2^ = .027
	Type of Script*Number of Scripts	*F*(1, 110) = 0.525, *p* = .470, η_p_^2^ = .005
	Aid*Number of Scripts	*F*(1, 110) = 0.567, *p* = .453, η_p_^2^ = .005
	Type of Script*Aid*Number of Scripts	*F*(1, 110) = 0.048, *p* = .086, η_p_^2^ = .000
A′	Type of Script	*F*(1, 110) = 0.703, *p* = .403, η_p_^2^ = .006
	Aid	*F*(1, 110) = 0.809, *p* = .370, η_p_^2^ = .007
	Number of Scripts	*F*(1, 110) = 0.974, *p* = .326, η_p_^2^ = .009
	Type of Script*Aid	*F*(1, 110) = 4.832, *p* = .030, η_p_^2^ = .042 *
	Type of Script*Number of Scripts	*F*(1, 110) = 0.012, *p* = .914, η_p_^2^ = .000
	Aid*Number of Scripts	*F*(1, 110) = 0.276, *p* = .601, η_p_^2^ = .002
	Type of Script*Aid*Number of Scripts	*F*(1, 110) = 0.051, *p* = .821, η_p_^2^ = .000
B′′_D_	Type of Script	*F*(1, 110) = 0.076, *p* = .783, η_p_^2^ = .000
	Aid	*F*(1, 110) = 1.570, *p* = .213, η_p_^2^ = .014
	Number of Scripts	*F*(1, 110) = 0.395, *p* = .531, η_p_^2^ = .004
	Type of Script*Aid	*F*(1, 110) = 0.207, *p* = .650, η_p_^2^ = .002
	Type of Script*Number of Scripts	*F*(1, 110) = 1.677, *p* = .198, η_p_^2^ = .015
	Aid*Number of Scripts	*F*(1, 110) = 3.378, *p* = .069, η_p_^2^ = .030
	Type of Script*Aid*Number of Scripts	*F*(1, 110) = 0.244, *p* = .623, η_p_^2^ = .002

*Note*:

* *p* < .05;

** *p* < .01;

*** *p* < .001

*Hits and false alarms*. For hits, we found a main effect of learning aid. Participants had a higher hit rate for translations (*M* = 0.73, *SD* = 0.13) than pictures (*M* = 0.70, *SD* = 0.12). No other differences were found. For false alarms, no main effects or interactions were found.

*A′*. A′ is an indicator of overall accuracy that is computed based on hits and false alarms. A′ ranges from 0 to 1, where 0 indicates very low accuracy and 1 very high accuracy. An A′ score of 0.5 indicates chance performance. An ANOVA with the A′ values showed no significant differences for the main effects of type of script, aid, or number of scripts, but the interaction of type of script by aid was significant. On the one hand, participants were more accurate in the Pinyin-translation condition (*M* = .82, *SD* = .10) than in the Pinyin-picture (*M* = .78, *SD* = .13), *t*(111) = 1.03, *p* = .026, *d* = .212. On the other hand, there were no differences between Hanzi-picture (*M* = .81, *SD* = .11) and Hanzi-translation (*M* = .80, *SD* = .13), *t*(111) = 1.16, *p* = .249, *d* = .111. Also, participants were more accurate for Hanzi-picture than for Pinyin-picture, *t*(111) = 2.06, *p* = .042, *d* = .194, while there were no differences between Hanzi-translation and Pinyin-translation. All the A′ scores were different from 0.5 (all *p* < .05).

In the yes/no recognition memory test, the words presented in Hanzi or Pinyin appeared without any learning aid. Thus, these results suggest that when learning was particularly challenging, as in the case of vocabulary learning in Hanzi, participants used all their resources to memorize it, and thus, there were no differences between the picture and translation conditions. However, when participants could read the word, as with Pinyin, translations seemed to be more effective.

*B′′*_*D*_. The B′′_D_ index is a measurement of the type of decision-making criteria adopted by participants. B′′_D_ scores nearing -1 indicate a lenient response criterion and a tendency to answer “yes”, while B′′_D_ scores closer to +1 show a stringent criterion and a tendency to answer “no”. As for B′′_D_, we found no significant differences for the main effects nor for their interactions. All of the scores were similar to zero (all *ps* > .05) except for pictures, *t*(111) = 2.36, *p* = .020, *d* = 0.22, for which participants applied a more stringent criterion (*M* = 0.087, *SD* = .39).

#### Memory in the word-aid recognition test

The word-aid recognition test was composed of four different types of pairs: Hanzi-translation, Hanzi-picture, Pinyin-translation, and Pinyin-picture. Half of the pairs were correct (same as presented in the study phase) and half were incorrect (never presented). See Table 5 for descriptive statistics and Table 6 for the full results of the ANOVAs for word-aid recognition analyses.

**Table 5:** Means (Standard deviations) for the proportions of hits, false alarms, A′ and B′′_D_ for the word-aid recognition test.

		Two-Scripts Participants		One-Script Participants	
		Picture	Translation	Picture	Translation
Hits	Hanzi	.78 (.15)	.80 (.13)	.80 (.16)	.81 (.13)
	Pinyin	.71 (.16)	.76 (.18)	.72 (.17)	.71 (.18)
False alarms	Hanzi	.22 (.16)	.27 (.20)	.25 (.16)	.27 (.17)
	Pinyin	.17 (.12)	.21 (.15)	.18 (.15)	.27 (.16)
*A′*	Hanzi	.86 (.09)	.84 (.13)	.85 (.10)	.84 (.11)
	Pinyin	.85 (.09)	.84 (.12)	.84 (.11)	.79 (.14)
*B′′*_*D*_	Hanzi	.01 (.52)	-.13 (.53)	-.13 (.49)	-.17 (.46)
	Pinyin	.26 (.50)	.07 (.52)	.26 (.46)	.01 (.53)

**Table 6:** Full results from the ANOVAs for the word-aid recognition test.

Hits	Type of Script	*F*(1, 110) = 21.489, *p <* .001, η_p_^2^ = .163 ***
	Aid	*F*(1, 110) = 1.729, *p* = .191, η_p_^2^ = .015
	Number of Scripts	*F*(1, 110) = 0.034, *p* = .855, η_p_^2^ = .000
	Type of Script*Aid	*F*(1, 110) = 0.039, *p* = .843, η_p_^2^ = .000
	Type of Script*Number of Scripts	*F*(1, 110) = 1.050, *p* = .308, η_p_^2^ = .009
	Aid*Number of Scripts	*F*(1, 110) = 2.534, *p* = .114, η_p_^2^ = .023
	Type of Script*Aid*Number of Scripts	*F*(1, 110) = 0.531, *p* = .468, η_p_^2^ = .005
False alarms	Type of Script	*F*(1, 110) = 13.256, *p <* .001, η_p_^2^ = .108 ***
	Aid	*F*(1, 110) = 13.446, *p <* .001, η_p_^2^ = .109 ***
	Number of Scripts	*F*(1, 110) = 1.782, *p* = .185, η_p_^2^ = .016
	Type of Script*Aid	*F*(1, 110) = 1.027, *p* = .313, η_p_^2^ = .009
	Type of Script*Number of Scripts	*F*(1, 110) = 0.564, *p* = .454, η_p_^2^ = .005
	Aid*Number of Scripts	*F*(1, 110) = 0.229, *p* = .633, η_p_^2^ = .002
	Type of Script*Aid*Number of Scripts	*F*(1, 110) = 2.079, *p* = .153, η_p_^2^ = .018
A′	Type of Script	*F*(1, 110) = 1.601, *p* = .208, η_p_^2^ = .014
	Aid	*F*(1, 110) = 6.377, *p* = .013, η_p_^2^ = .055 *
	Number of Scripts	*F*(1, 110) = 1.087, *p* = .300, η_p_^2^ = .010
	Type of Script*Aid	*F*(1, 110) = 0.435, *p* = .511, η_p_^2^ = .004
	Type of Script*Number of Scripts	*F*(1, 110) = 1.810, *p* = .181, η_p_^2^ = .016
	Aid*Number of Scripts	*F*(1, 110) = 1.185, *p* = .279, η_p_^2^ = .011
	Type of Script*Aid*Number of Scripts	*F*(1, 110) = 1.901, *p* = .171, η_p_^2^ = .017
B′′_D_	Type of Script	*F*(1, 110) = 27.601, *p <* .001, η_p_^2^ = .201 ***
	Aid	*F*(1, 110) = 13.243, *p <* .001, η_p_^2^ = .107 ***
	Number of Scripts	*F*(1, 110) = 1.134, *p* = .289, η_p_^2^ = .010
	Type of Script*Aid	*F*(1, 110) = 3.080, *p* = .082, η_p_^2^ = .027
	Type of Script*Number of Scripts	*F*(1, 110) = 0.399, *p* = .529, η_p_^2^ = .004
	Aid*Number of Scripts	*F*(1, 110) = 0.030, *p* = .863, η_p_^2^ = .027
	Type of Script*Aid*Number of Scripts	*F*(1, 110) = 1.081, *p* = .301, η_p_^2^ = .010

*Note*:

* *p* < .05;

** *p* < .01;

*** *p* < .001

*Hits and false alarms*. Participants had more hits for words presented in Hanzi (*M* = 0.80, *SD* = 0.11) than for Pinyin (*M* = 0.73, *SD* = 0.15). No other differences were found. For false alarms, we found more for Hanzi (*M* = 0.25, *SD* = .13) than for Pinyin (*M* = 0.21, *SD* = .12), and more for translations (*M* = 0.26, *SD* = 0.14) than for pictures (*M* = 0.21, *SD* = 0.12). No other differences were found.

*A′*. Analysis for A′ showed that participants were more accurate for word-pairs with pictures (*M* = 0.85, *SD* = 0.08) than with translations (*M* = 0.83, *SD* = 0.10). No other differences were found and all the scores were different from 0.5 (all *p* < .05).

*B′′*_*D*_. Participants applied a more lenient criterion for words presented in Hanzi (*M* = -0.11, *SD* = 0.39) than in Pinyin (*M* = 0.15, *SD* = 0.41), which explains the higher Hit and False alarm rates with that type of script. Also, participants applied a more lenient criterion for translations (*M* = -0.05, *SD* = 0.38) than pictures (*M* = 0.10, *SD* = 0.38). One sample t-tests for B′′_D_ against zero showed that participants applied a criterion different from a neutral one for Hanzi (*M* = -0.11, *SD* = 0.39), *t*(111) = -2.84, *p* = .005, *d* = -0.27, Pinyin (*M* = 0.15, *SD* = .41), *t*(111) = 3.93, *p* < .001, *d* = 0.37, pictures (*M* = 0.10, *SD* = 0.38), *t*(111) = 2.78, *p* = .006, *d* = 0.26, and also for Hanzi-translation (*M* = -0.15, *SD* = .49), *t*(111) = -3.19, *p* = .002, *d* = -.301, and Pinyin-picture (*M* = 0.26, *SD* = .47), *t*(111) = 5.84, *p* < .001, *d* = 0.55. For other conditions, participants’ criteria were neutral.

For translations, participants had a more lenient criterion than for pictures. This result, along with those for accuracy, suggests that a more lenient criterion for translations impaired accuracy for these word-pairs. Participants may have had the feeling that the words paired with translations were going to be better remembered, as estimated in their JOLs and reflected by their response criterion.

### Beliefs in learning

Our results did not show any significant differences for the main effects or interactions (see Table 7). Although we did not observe any differences in learners’ beliefs about foreign vocabulary learning in script nor in aid, we did observe significant differences in these variables in JOLs and memory. Thus, these results suggest that beliefs about the difficulty of a writing system or the effect of a learning aid are not linked to performance in memory or metamemory in this Chinese vocabulary learning task. Given the lack of empirical support in this section, we will not discuss this issue further.

**Table 7:** Mean (Standard deviation) for beliefs.

	Two-Scripts Participants		One-Script Participants	
	Picture	Translation	Picture	Translation
Hanzi	5.39 (2.00)	4.80 (1.99)	5.43 (2.13)	5.59 (2.39)
Pinyin	4.82 (2.14)	4.63 (2.26)	5.36 (2.49)	5.34 (2.51)

## Discussion

This research studied the effectiveness of different aids in foreign vocabulary learning for a non-alphabetic language such as Chinese in Russian learners, who are used to two scripts, and Colombian learners, who are used to only one script. We measured participants’ memory and metamemory for Chinese words, written either in Hanzi (logographic script) or Pinyin (alphabetic script), and paired with pictures and translations as learning aids. Overall, the main outcomes were that Pinyin was perceived as easier to learn than Hanzi, although there were no differences in memory. One-script users also judged pictures as better learning aids than translations, but two-scripts users did not. Again, these results were not aligned with actual memory performance. In the word-aid pair memory test, words paired with pictures were better identified. In general, these novel results contribute to understanding how learning a logographic script is perceived (JOLs) and how much it is actually learned (memory tests) by learners familiar with one or two different alphabetic scripts.

### Metamemory judgments for learning Chinese words

Pictures as learning aids increased JOLs only for two-script participants when learning Pinyin. No effect of learning aid in metamemory for Hanzi was found. Studies involving pictures in the monitoring of learning using JOLs [46, 49, 53] have found that pictures inflated the estimations about the future recallability of the words, but this metamemory pattern was not reflected in memory. However, other research on JOLs suggests that when the cognitive load of the task is high, some metamemory cues are ignored [55]. Learning Hanzi, a logographic script, seems to be particularly difficult for native speakers of alphabetic languages [54]. If processing Hanzi words caused a high cognitive load because they are perceived as complex drawings, that might have led participants to ignore previous beliefs about the effectiveness of pictures on memory.

Our results are not aligned with previous research results [53] reviewed in the Introduction. One explanation, apart from the cognitive load, is that their participants had a self-paced study time, whereas ours had a fixed time of five seconds. This time constraint might not have been enough for pictures to elicit a sense of fluency and, consequently, higher JOLs in this condition, but this experimental manipulation was necessary to minimize individual differences in reading time. Nonetheless, in a follow-up study by the same authors [49] participants had a set time of 6 seconds and found disparate results: Experiments 1 to 3 showed higher JOLs for pictures but no effect on memory, whereas in Experiment 4, after training in heuristics, JOLs became higher for translations but words were better remembered when paired with pictures. In sum, more research is needed to clarify further the effect of study time, of the heuristics about the predictability of JOLs, and the learning efficacy of aids in cross-script vocabulary learning.

In relation to the type of script, we confirmed our prediction of lower JOLs for words written in Hanzi, in accordance with the popular perception that Chinese characters are very difficult to learn because of their visual complexity [21, 71]. This result was expected also because participants were screened not to have previous experience with any similar script and artistic background, which could have given them some sense of familiarity or mastery.

We found two interactions in JOLs, involving the number of scripts learners are familiar with, which highlights the socio-linguistic environment’s relevance in the monitoring processes during second-language vocabulary learning. In relation to the interaction between the number of scripts and type of script, we found that two-script users did not show differences for JOLs for different scripts but that one-script users considered Pinyin (vs. Hanzi) as easier to learn. We reason that this is because when studying the monitoring process, it is important to consider the cue-familiarity heuristic in metacognition [71–73]. Two-script participants are used to two scripts, Latin and Cyrillic, and in addition, Russia has strong economic and cultural ties with China, which may expose Russians more to written Chinese (i.e., commercial products with labels in Chinese and interactions with visitors from China) compared to average Colombians. We suppose this factor may potentially boost a sense of familiarity for Russians towards the Chinese language and reduce the perceived difficulty of Hanzi when compared with Pinyin, but this supposition would warrant future research in sociolinguistics.

In line with the relevance of the socio-linguistic environment in the monitoring processes during foreign vocabulary learning, the perceived role of aids also differs depending on the country. Specifically, we found that one-script participants estimated the pictures as more effective for learning new words than translations, whereas these differences were not observed with two-script participants. Also, since Hanzi words are more complex than words in Pinyin, it is reasonable to think that our participants might have invested more time scanning words in Hanzi than in Pinyin. Hence, compared to the Pinyin condition, participants might have had less time to process the pictures in the Hanzi condition. Consequently, pictures in the Hanzi condition did not elicit high JOLs.

### Memory for learning Chinese words

Participants of this study completed two different types of recognition tests: a Chinese word recognition test, which provides information on the word forms that were learned, and a word-aid pair recognition test, which provides information about the word meanings that were learned.

Despite the to-be-learned words being presented only once and for a short period of time, this study detected conditions that improved learning. In the word recognition test, translations were more effective than pictures for learning Pinyin words, whereas in the word-aid recognition test, we found more hits and false alarms with Hanzi. However, that effect was not grounded in memory, but in the application of a more lenient criterion. This result is particularly intriguing because participants estimated worse recallability for Hanzi as reflected in JOLs. The perceived worse future memory for Hanzi could have pushed participants to invest more cognitive resources in memorizing it, making them believe they learned the words better and therefore resulting in the more lenient response criterion. Yet, these memory results are aligned with past research [64] that also found better memory of Hanzi words paired with definitions and not pictures in a study testing, as we did here, learners with an alphabetic native language, as we also hypothesized.

This last point strengthens what several authors have already proposed: there is a need to revise the current theories of word recognition. For example, the “dual route” model of word recognition [27], which is one of the most used theories in modern cognitive psychology, was mainly derived from research on English [74]. However, in the case of learning Chinese characters as beginners the phonological route is useless, unless the L1 of the learner was Japanese, that is, learners were already familiar with the writing system. Several authors point out that the research on CFL of non-phonographic languages may make a necessary and important contribution to widening the current theories of second language learning [1, 4].

In sum, these memory and metamemory results highlight an important issue: Participants were able to learn new words despite the short time of presentation and the complexity of the material. This finding is in line with studies showing that neural correlates of word-forms seem to appear as early as 200 ms after a single exposure [75]. In general, research on the brain basis of orthographic acquisition often fails to report behavioral results, most likely due to the particular encoding characteristics derived from methodology requirements. That is, that type of research is mainly focused on recording the differences at the brain level that not always are followed with the behavioral results.

Another important result is related to aids. The current literature on vocabulary learning in alphabetic languages finds support for the use of both pictures and translations. The present research explored aids in vocabulary learning in a logographic language and showed that while translations appear to be more effective for learning in the word recognition test (at least based on the results for Hits), pictures were more advantageous for learning in the word-aid test. Pictures being more effective for learning in the word-aid recognition test is a novel result, which most likely emerges from the divergences between our research design and previous ones. This leads us to strongly recommend future research to include different memory tests in order to properly understand which methods are more effective for vocabulary learning.

### Applied consequences

The outcomes of this research have potential applied consequences to second-language learning. First, metamemory measures are an informative and cost-effective means of evaluating the efficiency of the learning processes. A program for learning a new language can easily incorporate measures similar to JOLs or confidence. These measures not only offer valuable information on the subjective experience of participants and the factors and circumstances they think to help them to learn (so that they can be encouraged if correct, or challenged if incorrect), but they also may be helpful during the learning process. In that line, recent research has shown that the process of making JOLs can, under some circumstances, improve memory (see, for example [76]).

Second, this research showed that pictures are a more useful aid than translations to learn the meaning of a word in Hanzi, but the effect was mostly a response criterion one, and not a real memory advantage. That result, combined with the small advantage shown by presenting pictures, lead us to conclude it may not be worth searching for images representing concepts, which can be particularly challenging for abstract words. Second-language learning programs can adopt a more practical approach and use translations as more effective learning aids.

And, third, our results showed no large differences in memory between one-script and two-script users. Although it is relevant whether participants are familiar with the script of the new language [3], mastering one or two scripts does not seem to influence learning of a language in a different script or writing system. This would simplify the development of second-language programs for languages with different scripts, because they will not have to take into account the number of scripts students are used to.

### Limitations and future research

The present research has several limitations. First, we used only single Chinese-character concrete nouns in our experiment, but in real-life it is more common to find multi-character compound words. We opted for single characters because they allowed us to understand L2 Chinese vocabulary learning at the unit-level. We need further research to understand L2 Chinese vocabulary learning at more complex levels involving compound words, as well as words from other classes.

Second, written languages vary in their level of phonological access for learners, depending on the script of their mother tongue. Although we have revealed significant effects in learning Chinese words, it remains to be investigated whether research would show similar patterns in other phonologically inaccessible written languages for Latin or Cyrillic alphabets users, such as Arabic and Hebrew. To broadly understand the effects of pictures on foreign vocabulary learning, it would be necessary to conduct similar research involving other writing systems.

Third, the study time for words in our experiment was limited, as we chose to focus on the differences between scripts and aids. In real-life situations, learning is usually self-paced. Although our experiment offers valuable insight into the visual aids’ effectiveness in learning foreign words, we still need to address the potential imbalance in attentional distribution from the learners’ perspective. We encourage future experiments to allow participants to self-regulate study time, as this might be more precise in finding out the optimal mnemonic strategy in foreign vocabulary learning.

### Conclusions

The present study represents a significant contribution toward expanding our knowledge of the challenges that cross-script language learners encounter in the realm of second-language acquisition. First, it highlighted the interest and relevance of metamemory measures to study language learning. Second, it also highlighted the need for cross-script research to better understand language learning in a globalized world. It is our sincere hope that our findings have raised awareness about the complexities inherent in this process and will motivate further research in this area.
